# Effect of Heat Treatment on the Microstructure and Mechanical Properties of Mg-3.2Nd-2.5Gd-0.4Zn-0.5Zr (wt.%) Alloy

**DOI:** 10.3390/ma18235454

**Published:** 2025-12-03

**Authors:** Yao Li, Jingya Cui, Honghui Liu, Tong Mu, Lingyun An, Yongcai Zhang, Qiang Yu, Hailong Zhang, Xiushen Ye

**Affiliations:** 1 Qinghai Provincial Key Laboratory of Nanomaterials and Technology, School of Chemistry and Materials Science, Qinghai Minzu University, Xining 810007, China; ws2675331247@163.com (Y.L.); 18568968339@163.com (J.C.); 2 Key Laboratory of Green and High-End Utilization of Salt Lake Resources, Key Laboratory of Salt Lake Resources Chemistry of Qinghai Province, Qinghai Institute of Salt Lakes, Chinese Academy of Sciences, Xining 810008, China; liuhh@isl.ac.cn; 3 AECC South Industry Company Limited, Zhuzhou 412002, China; zyc230221@163.com (Y.Z.); yuqiang331@126.com (Q.Y.); zhl123456789@163.com (H.Z.)

**Keywords:** rare earth, Mg-Nd-Gd-Zn-Zr alloy, heat treatment, microstructure

## Abstract

**Highlights:**

**What are the main findings?**

- An optimal T6 treatment (520 °C × 10 h + 200 °C × 16 h) was established for the Mg-3.2Nd-2.5Gd-0.4Zn-0.5Zr alloy.

**What is the implication of the main finding?**

- The T6-treated alloy achieves a high-temperature UTS of 292 MPa at 150 °C while retaining high room-temperature strength.
- The enhanced strength and Brinell hardness primarily resulted from a high number density of β′ precipitates.

**Abstract:**

This study systematically examines the influence of heat treatment on the microstructure and mechanical properties of the Mg-3.2Nd-2.5Gd-0.4Zn-0.5Zr (wt.%) alloy using optical microscopy (OM), scanning electron microscopy (SEM), transmission electron microscopy (TEM), and mechanical testing. The as-cast alloy consists mainly of an α-Mg matrix and Mg_3_RE intermetallic phases. Solution treatment markedly improves microstructural homogeneity by dissolving most Mg-RE phases into the α-Mg matrix. Subsequent aging induces the formation of finely dispersed rare-earth precipitates, which contribute significantly to the improvement in hardness and strength. The optimal heat-treatment parameters are a solution treatment at 520 °C for 10 h followed by aging at 200 °C for 16 h (T6). After T6 treatment, the alloy exhibits an ultimate tensile strength (UTS) of 322 ± 2.0 MPa, a yield strength (YS) of 220 ± 23.0 MPa (increases of 53% and 88% relative to the as-cast alloy), and an elongation (EL) of 8.7 ± 0.2% at room temperature. At 150 °C, the UTS, YS, and EL reach 292 ± 2.6 MPa, 185 ± 1.1 MPa (41% and 62% improvements over the as-cast state), and 16 ± 1.0%, respectively, indicating excellent mechanical performance at elevated temperatures.

## 1. Introduction

Magnesium alloys, as the lowest-density structural metals currently utilized in engineering, exhibit a unique combination of low mass (1.738 g/cm^3^, nearly one-fifth that of steel and by a factor of two-thirds that of aluminum [1]), high specific strength, and elevated specific stiffness [2,3,4,5]. These attributes endow them with strategic importance and significant application potential in lightweight design for aerospace, transportation, and other fields [6,7]. Furthermore, due to their contribution to reducing energy consumption, magnesium alloys are acclaimed as the green engineering material of the 21st century [8]. However, the inherent limitations of pure magnesium—namely its low strength and poor ductility—significantly restrict its direct application in load-bearing structural components. To overcome these limitations, alloying and heat treatment have become essential strategies for improving its mechanical properties [9]. These methods aim to enhance magnesium’s performance by altering its microstructure and introducing new phases that can reinforce its matrix, especially at elevated temperatures.

Among the available strengthening routes, incorporating rare-earth (RE) elements is particularly effective, as RE additions can simultaneously activate multiple strengthening mechanisms—including solid-solution strengthening, precipitation hardening, dispersion strengthening, and grain refinement—thereby offering a comprehensive improvement in overall properties [10]. Substantial progress has been made in the development and engineering application of high-strength cast magnesium alloys [11], with several grades such as ZM6 (Mg-Nd-Zn-Zr system [12]) and GW103K (Mg-Gd-Y-Zr system) having been successfully developed. While traditional alloys represented by ZM6 have achieved practical application, their mechanical properties, corrosion resistance, and high-temperature stability still fall short of meeting the increasingly stringent requirements for advanced equipment such as aircraft [13]. The GW103K magnesium alloy utilizes Gd and Y as its primary alloying elements [14]. While the high Gd content enhances strength, it concurrently results in a significant loss of ductility [15,16]. Other existing high-strength cast magnesium alloys, such as WE43 (Mg-Y-RE-Zr), demonstrate good room-temperature strength, but the high chemical reactivity of Y can lead to the formation of oxide inclusions (e.g., Y_2_O_3_), compromising the performance consistency of cast components [17]. Although newer alloys like EV31A offer a better balance [18], there remains a pressing need for alloys that deliver superior strength without compromising ductility and high-temperature stability.

Therefore, the present work is motivated to develop a new Mg-RE alloy that strategically utilizes the complementary effects of Nd and Gd to achieve an optimal performance profile. The alloy design strategy centers on the synergistic addition of Nd, Gd, Zn, and Zr. Specifically, the primary rare-earth (RE) elements, Nd and Gd, are expected to enhance strength substantially via solid solution strengthening and, more importantly, through the formation of dense, fine-scale precipitates during subsequent aging treatment, which effectively strengthen the alloy by pinning dislocations [19]. Furthermore, it has been reported that certain RE elements can modify the c/a ratio of the HCP lattice and potentially activate non-basal slip systems, thereby improving plasticity [20]. From the perspective of solid solubility, Nd exhibits a maximum solid solubility in magnesium of only 3.7 wt.% (at 548 °C). The intermetallic compounds formed (Mg_41_Nd_5_, Mg_3_Nd, and MgNd [21]) provide limited strengthening effects. In contrast, Gd not only possesses significantly higher solid solubility (reaching 23.7 wt.% at 548 °C), but also exhibits a dramatic decrease to near zero at 150 °C. This characteristic enables the formation of various precipitates such as Mg_5_Gd and Mg_3_Gd, thereby facilitating effective precipitation strengthening [22,23]. The addition of Zn further enhances the age-hardening response by stabilizing the precipitate structure [24], while Zr acts as a potent grain refiner, concurrently improving strength and ductility according to the Hall-Petch relationship [25].

Solution and aging treatments are, therefore, essential thermal processes for tailoring the microstructure and mechanical properties of cast RE-containing magnesium alloys. Liuet al. [26] examined how different thermal treatment schedules influence the microstructure and mechanical behavior of a Mg-4Y-2Nd-1Gd-0.4Zr alloy. Their results indicated that subjecting the alloy to a solution treatment at 525 °C for 8 h, followed by aging at 225 °C for 16 h, enables the material to attain a tensile strength of 297 MPa. Their research also revealed differences in fracture mechanisms under different heat treatment conditions. Li et al. [27] systematically examines how the solution temperature governs the aging kinetics of a Mg_96.34_Gd_2.5_Zn_1_Zr_0.16_ alloy by tailoring its supersaturated solid solution. Their results demonstrated that the heat treatment regimen of 520 °C × 8 h (solution treatment) followed by 200 °C × 64 h (aging treatment) produced an exceptional ultimate tensile strength of 405 MPa, revealing the substantial impact of solution temperature on precipitation hardening effectiveness in the Gd-containing magnesium alloy system. Gu et al. [28] investigated the effect of double aging treatment on the microstructure and properties of a Mg-3Nd-1.5Gd-0.3Zn-0.5Zr alloy. In comparison with a conventional single-stage aging schedule, the double-aging route yields notable improvements in mechanical performance, elevating the UTS from 273 to 288 MPa and increasing the elongation from 4.9% to 6.6%, while simultaneously reducing the required aging duration to only 2 h.

Building upon these foundations, the present work systematically investigates the effect of heat treatment on the microstructure and mechanical properties of the newly designed Mg-3.2Nd-2.5Gd-0.4Zn-0.5Zr alloy, aiming to establish an optimal processing window for superior comprehensive performance.

## 2. Materials and Methods

The experimental alloy investigated in this work was an as-cast Mg-Nd-Gd-Zn-Zr rare-earth magnesium alloy commercially procured from Chongqing Yuhua New Material Technology Co., Ltd. (Chongqing, China). Alloy preparation used pure Mg (99.95 wt.%), Nd (99.5 wt.%), Gd (99.5 wt.%), Zn (99.5 wt.%), and Mg-30Zr master alloy. The raw materials were melted in an electrical resistance furnace and subsequently refined under a protective flux atmosphere. The melt was then cast into ingots measuring φ85 mm × 1000 mm using a semi-continuous casting procedure. The alloy’s chemical composition was quantified using inductively coupled plasma optical emission spectrometry (ICP-OES), and the measured values are presented in Table 1.

The microstructure of the alloy was tested with OM (Model 30XD-PC, Shanghai Optical Instrument Co., Ltd., Shanghai, China, SEM (Model Apreo 2s, Thermo Fisher Scientific Inc., Waltham, MA, USA), and high-resolution transmission electron microscope (Tecnai G2 F20, FEI, Hillsboro, OR, USA). An Oxford energy-dispersive X-ray spectrometer (EDS) (Oxford Instruments, Abingdon, UK) was employed to examine the elemental distribution and phase composition. A stepwise grinding procedure was carried out on the metallographic specimens with SiC papers from coarse (240 grit) to fine (5000 grit), subjected to mechanical polishing using YMP-2 polishing machine (Shangahi Metallurgical Equipment Company LTD., Shanghai, China), and subsequently etched with a 4 vol.% nitric acid ethanol solution to reveal the microstructure. Phase constituents were determined using a D8 DISCOVER X-ray diffractometer (BRUKER, Karlsruhe, Germany). Brinell hardness tests were conducted on a THBS-62.5 hardness tester (Beijing Time High Technology CO., LTD., Beijing, China) with a load of 10 kg and a dwell time of 30 s. Each reported hardness value represents the mean of quintuplicate measurements, with the standard deviation being less than ±2.5 HBW. Differential scanning calorimetry (DSC) was conducted using an STA509 synchronous thermal analyzer (NETZSCH, Brvaria, Germany). The sample was heated up to 650 °C at 10 °C/min and subsequently cooled to 50 °C at the same rate, with the entire process conducted under a protective argon atmosphere.

**Figure 1:**
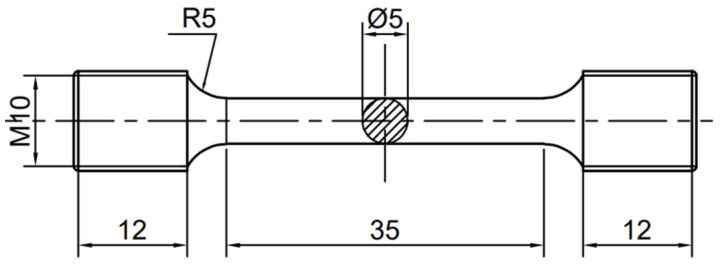
Dimensions of the cylindrical tensile specimen.

A series of solution treatments were conducted on the as-cast specimens, employing temperatures of 510, 520, and 530 °C with corresponding durations of 6, 10, and 14 h, respectively, then water quenching. Subsequently, samples solution-treated at 520 °C for 10 h were aged at 175, 200, and 225 °C for various durations up to 130 h. Figure 1 presents the detailed geometry of the rod-shaped tensile specimens. Tensile properties were obtained from tests on at least three parallel specimens.

## 3. Results and Discussion

### 3.1. Microstructural Observation of As-Cast Alloy

**Figure 2:**
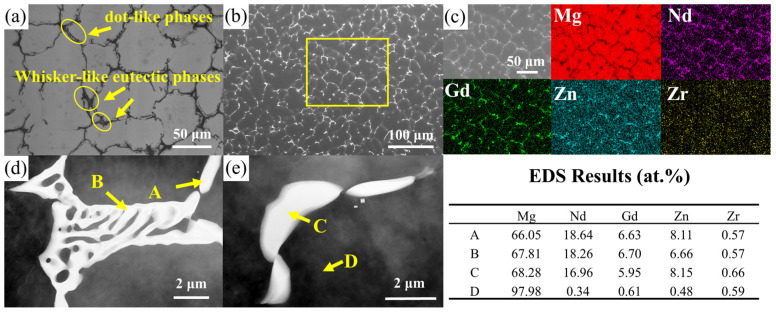
Microstructure of the as-cast experimental alloy: (**a**) OM image; (**b**) SEM image; (**c**) Local magnification of the rectangular region in (**b**) and corresponding EDS elemental mapping; (**d**) TEM image of the network phase; (**e**) TEM image of the dot-like phase.

Figure 2 presents the OM, SEM, and TEM images of the as-cast alloy. All as-cast alloys exhibit a dendritic microstructure, consisting of equiaxed α-Mg grains, non-continuous whisker-like eutectic phases located in interdendritic regions and along grain boundaries, and dot-like phases distributed in the grain interiors. The mean diameter of the α-Mg grains, as measured by the intercept method, is 27.25 μm. Figure 2b shows the SEM image of the as-cast alloy, revealing a continuous network of secondary phases aligned with the grain boundaries. The secondary phases distributed along grain boundaries and within grain interiors have a characteristic size on the order of 20–30 μm and occupy approximately 6.29 vol.% of the as-cast microstructure. To determine the elemental content of the secondary phases, EDS elemental mapping was performed. The mapping results in Figure 2c reveal that the matrix is predominantly composed of Mg with trace amounts of Zr, while the rare earth elements Nd and Gd are primarily segregated along the grain boundaries. To determine the phase composition of the experimental alloy, phase analysis was conducted on the as-cast material. There are significant discrepancies regarding the Mg-Nd-Gd-Zn-Zr alloys’ as-cast microstructure in current research. Yang et al. [29] propose that the as-cast alloy primarily composes of an α-Mg matrix, networked Mg_3_RE intermetallic phases distributed along grain boundaries, and short lath-shaped Mg_12_Nd phases within grain interiors. Su et al. [30], however, contend that the as-cast microstructure primarily consists of an α-Mg matrix with Mg_12_Nd eutectic phases along grain boundaries. In contrast, A. Kielbus [31] suggest that the as-cast alloy is mainly consisted of a magnesium matrix and Mg_12_(Nd_x_Gd_1−x_) phases precipitated along grain boundaries. They propose that Mg_12_(Nd_x_Gd_1−x_) is derived from the Mg_12_Nd phase through substitution of Nd by Gd atoms, which is facilitated by their similar atomic radii, allowing Gd to replace Nd without disrupting the original crystal structure.

**Figure 3:**
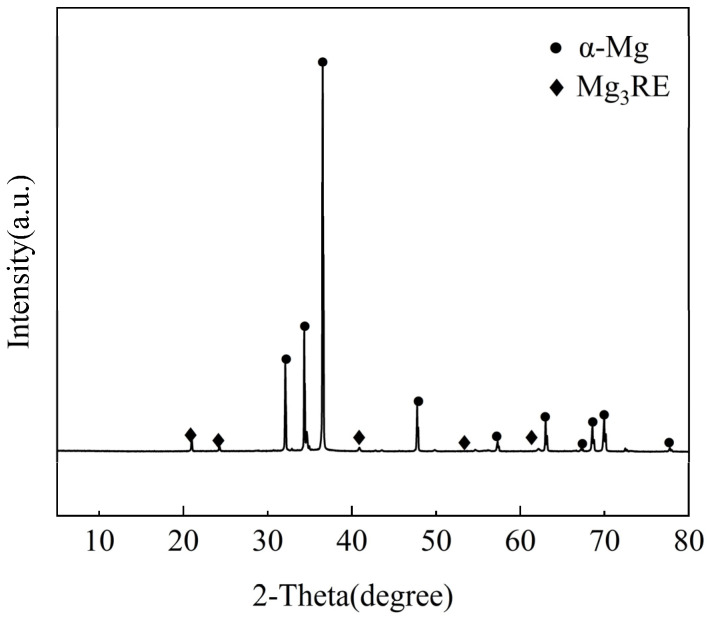
XRD of the as-cast experimental alloy.

In the present study, XRD results (as shown in Figure 3) indicate that all diffraction peaks, apart from those corresponding to α-Mg, can be indexed to the Mg_3_RE phase. This is consistent with the predictions from the Mg-Nd and Mg-Gd phase diagram, which shows that at high temperatures, Mg_3_RE intermetallic compounds are expected to form, particularly in the non-equilibrium solidification process [1]. However, this does not rule out the possibility that the content of the lath-shaped phase is below the detection limit of the XRD technique. To further confirm the chemical constituent of the secondary phases, EDS point analysis was performed. The network phase attached to the grain boundary, indicated by arrow A in Figure 2d, was confirmed by EDS analysis with atomic percentages of 66.05% Mg, 18.64% Nd, and 6.63% Gd. The resulting Mg/(Nd + Gd) atomic ratio is approximately 3:1, which, combined with the XRD results, confirms this phase as Mg_3_RE. The dot-like phases observed in Figure 2a are actually consisted of smaller blocky precipitates when viewed at higher magnification, according to Figure 2e. EDS analysis performed at the location marked by arrow C in Figure 2e confirmed that the phase is Mg_3_RE.

Based on the thermodynamic data from binary phase diagrams at the Mg-rich end for the Gd-Mg and Nd-Mg systems, both systems exhibit eutectic reactions with a eutectic temperature of 548 °C [1]. During solidification, the primary α-Mg phase forms first, gradually enriching the remaining liquid with solute elements. When the alloy temperature rises toward the eutectic region, the locally enriched Gd and Nd atoms near the solid–liquid interface participate in eutectic transformations, leading to the generation of Mg_3_RE compounds. This process ultimately causes rare-earth elements to segregate along the grain boundaries.

### 3.2. Solution Treatment

**Figure 4:**
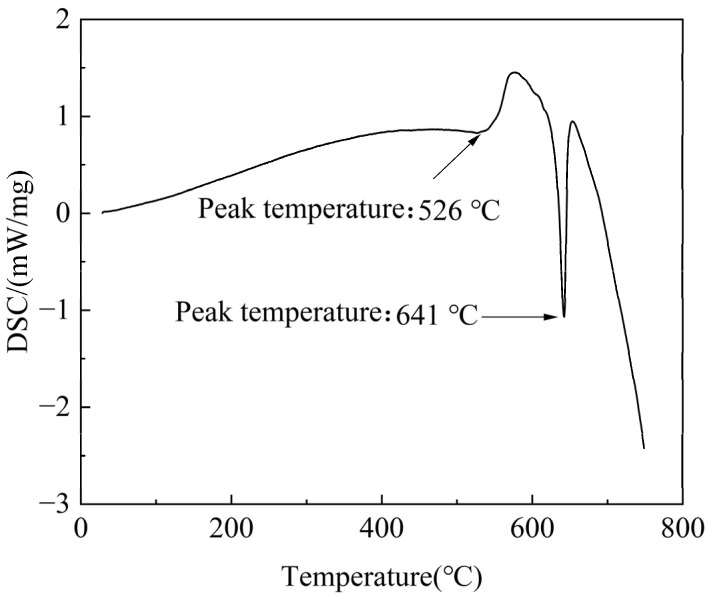
DSC curve of the as-cast experimental alloy.

To determine appropriate solution treatment parameters, DSC analysis was performed on the as-cast alloy, with the resultant curve presented in Figure 4. Two endothermic peaks are observed at 526 °C and 641 °C, which are attributed to the melting of secondary phases and the Mg matrix during the heating procedure, respectively. Based on the DSC results, the solution treatment parameters for the experimental alloy were selected within the ranges of 510–530 °C and 6–14 h, respectively, as listed in Table 2. The optimal solution treatment parameters were determined by systematically evaluating the dissolution behavior of secondary phases into the α-Mg matrix.

**Figure 5:**
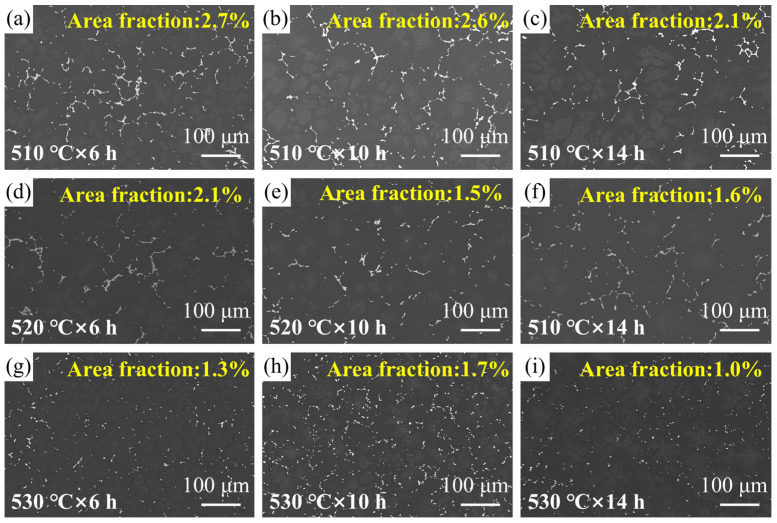
SEM images of the alloy after solution treatment under different states. Area fraction of secondary phases for each condition is inset in (**a**–**i**).

**Figure 6:**
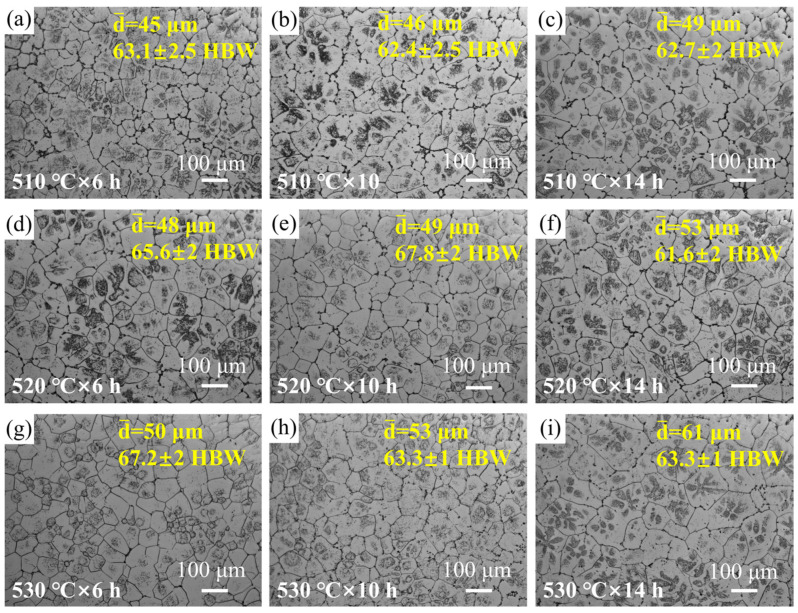
OM images of the alloy after solution treatment under different states. The corresponding average grain size $\overset{-}{d}$ and Brinell hardness value for each condition is inset in (**a**–**i**).

Figure 5 shows SEM images of the experimental alloy after solution treatment. It can be observed that the secondary phases attached to the grain boundaries have undergone varying degrees of dissolution. When the solution temperature was 510 °C, a considerable amount of continuous secondary phases still remained at the grain boundaries even when the solution time was extended from 6 h to 14 h. Therefore, the 510 °C solution temperature was excluded from consideration. When the solution treatment temperature was raised to 520 °C, the amount of secondary phases located at the grain boundaries was significantly reduced compared to those observed at 510 °C. When the solution temperature was further raised to 530 °C, the continuous network of secondary phases along the grain boundaries completely dissolved. However, as evidenced in Figure 6, this condition induces significant grain growth, which is detrimental to mechanical properties. Thus, the optimal solution treatment condition for the alloy is determined to be 520 °C for 10 h.

### 3.3. Age Hardening Behavior and Precipitate

**Figure 7:**
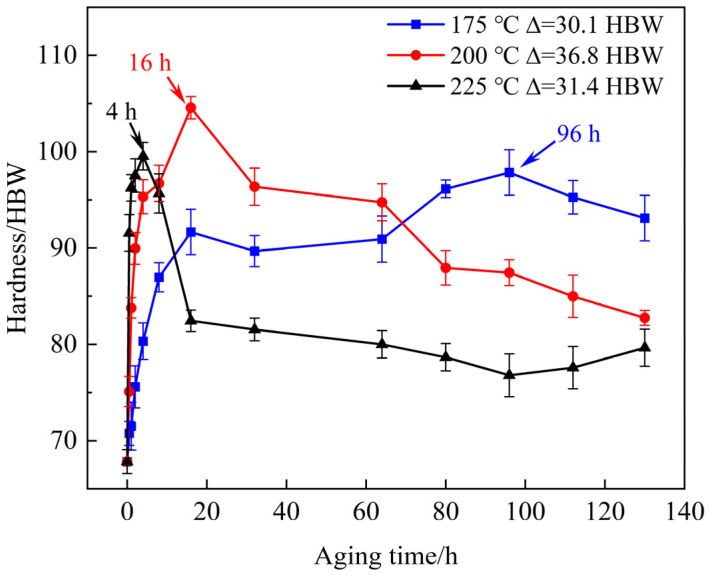
Aging-hardness response after solution treatment at 520 °C for 10 h under aging temperatures of 175~225 °C.

Following solution treatment at 520 °C for 10 h, the experimental alloy specimens were subjected to aging treatment at three different temperatures (175 °C, 200 °C, and 225 °C) for durations ranging from 0.5 h to 130 h. Figure 7 illustrates the age-hardening response of the experimental alloy at above three temperatures. During aging at 225 °C, the hardness rises rapidly to a peak value of 99.54 ± 1.4 HBW within just 4 h, after which it gradually decreases with further prolongation of aging time. At 200 °C, the hardness increases more moderately and reaches the highest peak value of 104.56 ± 1.2 HBW after 16 h of aging, followed by a slight decline at longer times. In contrast, aging at 175 °C resulted in a gradual increase in Brinell hardness, attaining a peak value of 97.9 ± 2.4 HBW after 96 h. This trend reflects the strong temperature dependence of precipitation kinetics: higher aging temperatures accelerate β′ precipitation and shorten the time to peak hardness but also lead to earlier over-aging, whereas lower temperatures retard both precipitation and over-aging. Considering the balance between time efficiency and performance, the optimum heat treatment regime for the experimental alloy was identified as solution treatment at 520 °C for 10 h then aging at 200 °C for 16 h.

**Figure 8:**
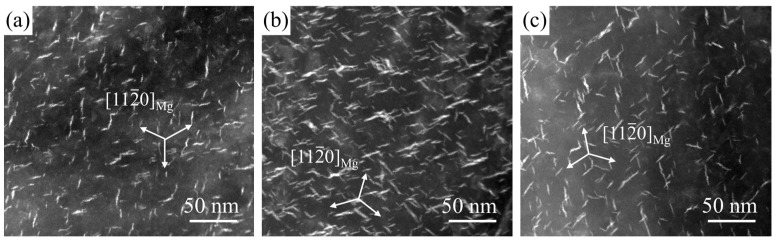
HAADF-STEM Images illustrating the distribution of β′ phase in the experimental alloy at peak aging. (**a**) 175 °C × 96 h; (**b**) 200 °C × 16 h; (**c**) 225 °C × 4 h.

Figure 8 presents HAADF-STEM images of the precipitates in the experimental alloy under three peak-aged conditions, viewed along the [11$\overset{-}{2}$0]_Mg_ zone axis. In all conditions, the precipitates are uniformly distributed within the α-Mg matrix, exhibiting a short rod-like morphology. The precipitates have a length of 10–18 nm and a thickness of 3 nm. The observed multiple precipitate variants originate from their formation on the three equivalent {11$\overset{-}{2}$0} prismatic planes of the HCP matrix [32]. The bright contrast in the HAADF-STEM images confirms the enrichment of Nd, Gd, and Zn (elements with high atomic numbers) within these precipitates. At the same magnification, it is clearly observed that the sample peak-aged at 200 °C exhibits a higher number density of β′ precipitates than those treated at the other temperatures. Quantitative analysis of the β′ precipitate number density cannot be accurately determined from the HAADF-STEM images alone, as these projection images contain precipitates located at different depths within the foil thickness. However, a rough quantification based on Figure 8 indicates that the sample peak-aged at 200 °C contains approximately twice the number density of β′ precipitates compared to the 175 °C × 96 h sample and 2.5 times that of the 225 °C × 4 h sample. Furthermore, the β′ precipitates in the 225 °C peak-aged condition appear coarser and less uniformly distributed, indicating partial coarsening at this higher temperature. The significantly higher number density of fine β′ precipitates at 200 °C correlates directly with the superior hardness observed in this peak-aged state, confirming that precipitation hardening is the dominant strengthening mechanism.

The age-hardening response is primarily attributed to the precipitation of β′ phases, which form as fine plates on the prismatic planes of the α-Mg matrix. This observation is consistent with the well-established precipitation sequence for Mg-Nd-Gd-based alloys: supersaturated solid solution (SSSS) → GP zones → β″ → β′ → β_1_ → β (Mg_41_RE_5_). At early aging stages, GP zones and β″ precursors contribute modestly to strengthening. As aging progresses toward the peak-aged condition, a high number density of fine β′ precipitates develop, effectively pinning dislocations and giving rise to the maximum hardness. With further aging beyond the peak, these β′ precipitates coarsen and/or transform into more stable β_1_ and β equilibrium phases, reducing their strengthening efficiency and resulting in the observed over-aging. The formation of the metastable β′ phase, rather than the equilibrium Mg_41_RE_5_ phase, at the peak-aged condition is expected, because the phase diagram predicts Mg_41_RE_5_ to be stable only under equilibrium conditions, whereas the precipitation sequence is kinetically controlled [29]. The β′ precipitate morphology and number density observed in the peak-aged samples (Figure 8) therefore provide direct microstructural evidence for the hardness evolution shown in Figure 7 and demonstrate the effectiveness of the applied T6 treatment in enhancing strength through precipitation hardening.

### 3.4. Characterization of Mechanical Properties and Fracture Surface Morphology

**Figure 9:**
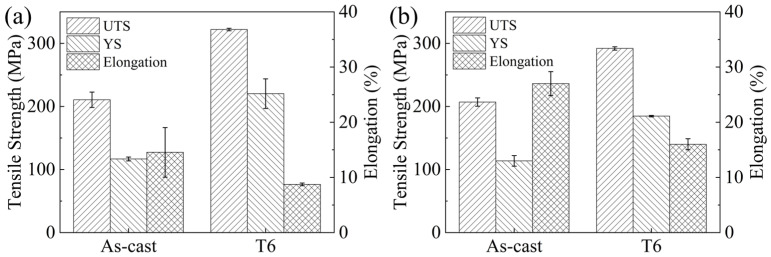
Mechanical Properties of the Experimental Alloy. (**a**) At room temperature; (**b**) At an elevated temperature of 150 °C.

Figure 9 shows the mechanical properties of the experimental alloy at room temperature and elevated temperatures under both as-cast and T6-treated conditions, the corresponding mechanical properties are presented in Table 3. The as-cast experimental alloy exhibits UTS, YS, and EL of 210 ± 12 MPa, 117 ± 3 MPa, and 14.5 ± 4.5% at room temperature, respectively. When tested at an elevated temperature of 150 °C, the corresponding values are 207 ± 6.5 MPa, 114 ± 8.3 MPa, and 27 ± 2.1%. After T6 treatment, the alloy achieves UTS, YS, and EL of 322 ± 2 MPa, 220 ± 23 MPa, and 8.7 ± 0.2% at room temperature, and 292 ± 2.6 MPa, 185 ± 1.1 MPa, and 16 ± 1% at 150 °C, respectively.

Tensile properties of the as-cast alloy at room temperature are governed by the grain size and the distribution characteristics of the secondary phases-namely, their size, morphology, and distribution along grain boundaries and inside the grain interiors [33,34,35]. The Mg_3_RE eutectic phase along the grain boundaries of the as-cast alloy tends to fracture readily during tensile testing due to the development of localized stress concentration, resulting in a detrimental impact on the alloy’s mechanical performance. After heat treatment, rod-shaped β′ precipitates are formed within the Mg matrix, which effectively impede dislocation glide. Consequently, the β′ phase is identified as the principal strengthening phase responsible for the high strength of T6-treated alloys [36].

Under high-temperature tensile conditions, grain boundaries act as preferential sites for strain accommodation, with grain boundary sliding emerging as an additional accommodated deformation mechanism [37]. In the experimental alloy, the Mg_3_RE phase located at grain boundaries is recognized as a thermally stable phase. Thus, it effectively suppresses grain boundary sliding and thereby enhances the overall mechanical stability of the boundaries at elevated temperatures. Simultaneously, the densely arranged β′ precipitates strongly inhibit dislocation glide on basal planes. Owing to these combined strengthening effects, the T6-treated experimental alloy exhibits high strength at elevated temperatures, achieving an UTS of 292 ± 2.6 MPa, which demonstrates its excellent high-temperature performance.

**Figure 10:**
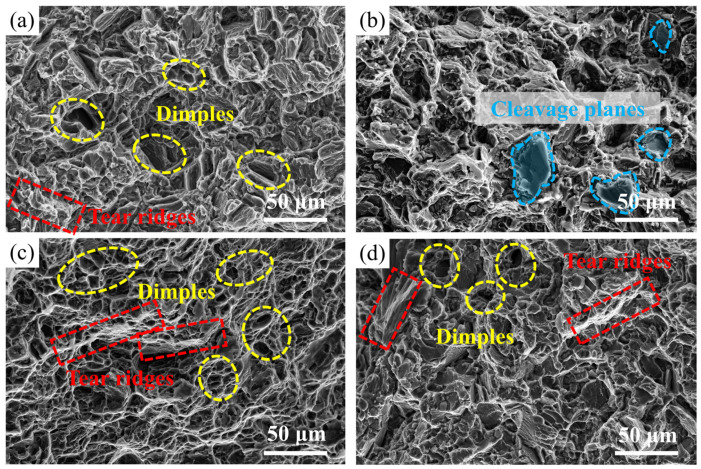
Tensile Fracture Morphologies of the Experimental Alloy. (**a**) As-cast alloy tested at room temperature; (**b**) T6-treated alloy tested at room temperature; (**c**) As-cast alloy tested at 150 °C; (**d**) T6-treated alloy tested at 150 °C.

Figure 10 shows the tensile fracture surfaces of the as-cast and T6-treated experimental alloys tested at room temperature and 150 °C. The tensile fracture surface of the as-cast alloy at room temperature (Figure 10a) exhibits characteristic dimples and tear ridges, indicative of ductile fracture, which is consistent with its high elongation of 14.5%. In this condition, the matrix is comparatively soft and the deformation is mainly accommodated by microvoid nucleation, growth, and coalescence, with the coarse Mg_3_RE phases at grain boundaries acting as preferential void nucleation sites. In contrast, the T6-treated alloy under the same conditions (Figure 10b) shows numerous cleavage facets and river patterns, revealing a quasi-cleavage or brittle-like fracture behaviour. This abrupt change in fracture mode is the combined result of the intrinsic crystallography of Mg, the addition of alloying elements (Nd, Gd, Zn, and Zr), and the T6-modified microstructure. On the one hand, the HCP structure of Mg inherently provides only a limited number of independent slip systems at room temperature, which restricts plastic deformation. On the other hand, in the T6 condition, a high density of fine β′ precipitates is uniformly dispersed within the matrix. These precipitates strongly pin dislocations and severely impede their motion, leading to pronounced local stress concentration. Because the limited plasticity of the HCP Mg matrix prevents efficient stress relaxation via the activation of sufficient non-basal slip systems, the local stress can readily exceed the cleavage strength, thereby triggering cleavage crack initiation and propagation and resulting in the macroscopically brittle fracture features observed. The tensile fracture surfaces of both the as-cast and T6-treated alloys tested at 150 °C (Figure 10c,d) are characterized by abundant dimples and pronounced ductile fracture features, which are consistent with their increased elongation values at elevated temperature. At 150 °C, the higher testing temperature activates additional non-basal slip systems in the HCP Mg matrix and enhances dislocation mobility. Under these conditions, dislocations can bypass or cut through the precipitates more readily, for example, by cross-slip or non-basal slip, which mitigates the severe stress concentration that occurs at room temperature. As a result, even in the T6-treated alloy a more favorable balance between precipitate strengthening and matrix deformability is achieved, and fracture proceeds predominantly through microvoid nucleation, growth, and coalescence rather than cleavage. These observations confirm that the transition from brittle-like fracture at room temperature to ductile fracture at 150 °C is not caused by a single factor, but arises from the combined effects of the HCP crystal structure of Mg, the presence and distribution of β′ precipitates, and the temperature-dependent activation of additional deformation modes.

From an application-oriented perspective, the T6 treatment (520 °C/10 h solution treatment followed by aging at 200 °C/16 h) is recommended as the optimal processing route for the Mg-3.2Nd-2.5Gd-0.4Zn-0.5Zr alloy. This regimen produces a high number density of fine β′ precipitates, resulting in an excellent combination of tensile strength and ductility at both room temperature and 150 °C. Compared to commercial Mg-RE alloys that rely on high Gd (e.g., GW103K) or Y (e.g., WE43), the present alloy achieves comparable high-temperature strength with a substantially lower total rare-earth content. This, combined with the simplified single-stage aging process, makes this alloy and its associated heat treatment a promising and cost-efficient solution for lightweight structural components in aerospace and automotive applications.

## 4. Conclusions

The Mg-3.2Nd-2.5Gd-0.4Zn-0.5Zr (wt.%) alloy was comprehensively examined in both the as-cast state and after heat treatment to evaluate its microstructural evolution and mechanical performance. The principal findings of this work are outlined below:

1. The as-cast microstructure composes of an α-Mg matrix with Mg_3_RE phases distributed along grain boundaries and within grain interiors. The average grain size was measured to be 27.25 μm.
2. Based on systematic hardness measurements, mechanical property testing, and microstructural characterization of the T6-treated alloy, the optimal heat treatment regime was established as solution treatment at 520 °C for 10 h then aging at 200 °C for 16 h.
3. After T6 treatment, the experimental alloy achieves UTS and YS of 322 ± 2.0 MPa and 220 ± 23.0 MPa at room temperature, representing increases of 53% and 88%, respectively, compared with the as-cast condition, with an EL of 8.7 ± 0.2%. When tested at 150 °C, the UTS and YS reach 292 ± 2.6 MPa and 185 ± 1.1 MPa (increases of 41% and 62% over the as-cast alloy), accompanied by an EL of 16 ± 1.0%.
4. The fracture mode of the Mg-3.2Nd-2.5Gd-0.4Zn-0.5Zr alloy exhibits significant variations with heat treatment condition and testing temperature. At room temperature, the as-cast alloy undergoes ductile fracture with dimples, whereas the T6-treated alloy displays quasi-cleavage brittle fracture due to the strong pinning effect of fine β′ precipitates. At 150 °C, both the as-cast and T6-treated alloys transition to predominantly ductile fracture characterized by abundant dimples, where thermal activation at elevated temperature enhances plasticity by facilitating additional slip.

## Figures and Tables

**Table 1:** Actual chemical composition of the as-cast experimental alloy (wt.%).

Nd	Gd	Zn	Zr	Mg
3.21	2.52	0.37	0.49	Bal.

**Table 2:** Solution treatment temperature and time for the experimental alloy.

Serial Number	Temperature/°C	Time/h
a	510	6
b	510	10
c	510	14
d	520	6
e	520	10
f	520	14
g	530	6
h	530	10
i	530	14

**Table 3:** Mechanical Properties of the Experimental Alloy at Different Test Temperatures.

Experimental Alloy	Testing Temperature	UTS (MPa)	YS (MPa)	EL (%)
As-cast	Room temperature	210 ± 12.0	117 ± 3.0	14.5% ± 4.5
T6-treated	Room temperature	322 ± 2.0	220 ± 23.0	8.7% ± 0.2
As-cast	150 °C	207 ± 6.5	114 ± 8.3	27% ± 2.1
T6-treated	150 °C	292 ± 2.6	185 ± 1.1	16% ± 1.0

## Data Availability

The original contributions presented in this study are included in the article. Further inquiries can be directed to the corresponding authors.

## References

[1] Meier J.M., Caris J., Luo A.A. (2022). Towards high strength cast Mg-RE based alloys: Phase diagrams and strengthening mechanisms. J. Magnes. Alloys.

[2] Li J.H., Jie W.Q., Yang G.Y. (2008). Effect of gadolinium on aged hardening behavior, microstructure and mechanical properties of Mg-Nd-Zn-Zr alloy. Trans. Nonferr. Met. Soc. China.

[3] Pan F.S., Yang M.B., Chen X.H. (2016). A Review on Casting Magnesium Alloys: Modification of Commercial Alloys and Development of New Alloys. J. Mater. Sci. Technol..

[4] Deng Q.C., Chang Z.Y., Su N., Luo J., Liang Y.Y., Jin Y.H., Wu Y.J., Peng L.M., Ding W.J. (2025). Developing a novel high-strength Mg-Gd-Y-Zn-Mn alloy for laser powder bed fusion additive manufacturing process. J. Magnes. Alloys.

[5] Yang Y., Xiong X.M., Chen J., Peng X.D., Chen D.L., Pan F.S. (2021). Research advances in magnesium and magnesium alloys worldwide in 2020. J. Magnes. Alloys.

[6] Pollock T.M. (2010). Weight loss with magnesium alloys. Science.

[7] Kumar D.S., Sasanka C.T., Ravindra K., Suman K.N.S. (2015). Magnesium and its alloys in automotive applications—A review. Am. J. Mater. Sci. Technol..

[8] Xu T.C., Yang Y., Peng X.D., Song J.F., Pan F.S. (2019). Overview of advancement and development trend on magnesium alloy. J. Magnes. Alloys.

[9] Fan R., Wang L., Zhao S., Wang L., Guo E. (2023). Strengthening of Mg Alloy with Multiple RE Elements with Ag and Zn Doping via Heat Treatment. Materials.

[10] Xie H.B., Pan H.C., Ren Y.P., Sun S., Wang L.Q., He Y.F., Qin G.W. (2018). Co-existences of the two types of β’ precipitations in peak-aged Mg-Gd binary alloy. J. Alloys Compd..

[11] Ping D.H., Hono K., Nie J.F. (2003). Atom probe characterization of plate-like precipitates in a Mg–RE–Zn–Zr casting alloy. Scr. Mater..

[12] Fu J., Chen S. (2021). Microstructure Evolution and Mechanical Properties of As-Cast and As-Compressed ZM6 Magnesium Alloys during the Two-Stage Aging Treatment Process. Materials.

[13] Dang C., Wang J.F., Wang J.X., Yu D., Zheng W.X., Xu C.B., Lu R.P. (2023). Effect of lamellar LPSO phase on mechanical properties and damping capacity in cast magnesium alloys. J. Mater. Res. Technol..

[14] Liao H.G., Fu P.H., Peng L.M., Li J., Zhang S.Q., Hu G.Q., Ding W.J. (2017). Microstructure and mechanical properties of laser melting deposited GW103K Mg-RE alloy. Mater. Sci. Eng. A.

[15] Chen C., Han D., Wang M., Cai T., Liang N., Beausir B., Liu H., Yang S. (2022). The Effect of Rotary-Die Equal-Channel Angular Pressing Process on the Microstructure, the Mechanical and Friction Properties of GW103 Alloy. Materials.

[16] Li M., Yao M., Liu L., Zhang X., Xing Z., Xia X., Liu P., Wan Y., Chen Q., Wang H. (2024). Effects of an LPSO Phase Induced by Zn Addition on the High-Temperature Properties of Mg-9Gd-2Nd-(1.5Zn)-0.5Zr Alloy. Materials.

[17] Luo K., Zhang L., Wu G.H., Liu W.C., Ding W.J. (2019). Effect of Y and Gd content on the microstructure and mechanical properties of Mg–Y–RE alloys. J. Magnes. Alloys.

[18] Jana A., Das M., Balla V.K. (2020). Effect of heat treatment on microstructure, mechanical, corrosion and biocompatibility of Mg-Zn-Zr-Gd-Nd alloy. J. Alloys Compd..

[19] He S.M., Zeng X.Q., Peng L.M., Gao X., Nie J.F., Ding W.J. (2007). Microstructure and strengthening mechanism of high strength Mg–10Gd–2Y–0.5Zr alloy. J. Alloys Compd..

[20] Sandlöbes S., Zaefferer S., Schestakow I., Yi S., Gonzalez-Martinez R. (2011). On the role of non-basal deformation mechanisms for the ductility of Mg and Mg–Y alloys. Acta Mater..

[21] Nie J.F. (2012). Precipitation and Hardening in Magnesium Alloys. Metall. Mater. Trans. A.

[22] Xie J.S., Zhang J.H., You Z.H., Liu S.J., Guan K., Wu R.Z., Wang J., Feng J. (2021). Towards developing Mg alloys with simultaneously improved strength and corrosion resistance via RE alloying. J. Magnes. Alloys.

[23] Xie H., Wu G.H., Zhang X.L., Liu W.C., Ding W.J. (2021). The role of Gd on the microstructural evolution and mechanical properties of Mg-3Nd-0.2Zn-0.5Zr alloy. Mater. Charact..

[24] Ding Z.B., Zhao Y.H., Lu R.P., Yuan M.N., Wang Z.J., Li H.J., Hou H. (2019). Effect of Zn addition on microstructure and mechanical properties of cast Mg-Gd-Y-Zr alloys. Trans. Nonferr. Met. Soc. China.

[25] Qian M., Das A. (2006). Grain refinement of magnesium alloys by zirconium: Formation of equiaxed grains. Scr. Mater..

[26] Liu Z.J., Wu G.H., Liu W.C., Pang S., Ding W.J. (2012). Effect of heat treatment on microstructures and mechanical properties of sand-cast Mg–4Y–2Nd–1Gd–0.4Zr magnesium alloy. Trans. Nonferr. Met. Soc. China.

[27] Li J.C., He Z.L., Fu P.H., Wu Y.J., Peng L.M., Ding W.J. (2016). Heat treatment and mechanical properties of a high-strength cast Mg–Gd–Zn alloy. Mater. Sci. Eng. A.

[28] Gu K., Zeng X.Q., Chen B., Wang Y.X. (2021). Effect of double aging on mechanical properties and microstructure of EV31A alloy. Trans. Nonferr. Met. Soc. China.

[29] Yang H., Zander D., Jiang B., Huang Y., Gavras S., Kainer K.U., Dieringa H. (2020). Effects of heat treatment on the microstructural evolution and creep resistance of Elektron21 alloy and its nanocomposite. Mater. Sci. Eng. A.

[30] Su X., Feng Z.J., Huang J.F., Du X.D., An R.S., Wang F., Lou Y.C. (2021). Influence of a low-frequency alternating magnetic field on hot tearing susceptibility of EV31 magnesium alloy. China Foundry.

[31] Kiełbus A. (2007). Microstructure and mechanical properties of Elektron 21 alloy after heat treatment. J. Achiev. Mater. Manuf. Eng..

[32] Saito K., Hiraga K. (2011). The Structures of Precipitates in an Mg-0.5 at%Nd Age-Hardened Alloy Studied by HAADF-STEM Technique. Mater. Trans..

[33] Wu Q.Y., Wu Y.J., Deng Q.C., Ding C.Y., Zhang Y., Peng N.X., Jia L.C., Chang Z.Y., Peng L.M. (2024). Effect of Gd content on microstructure and mechanical properties of Mg-xGd-Zr alloys via semicontinuous casting. J. Magnes. Alloys.

[34] Chen H.C., Wen H.B., Sun J.L., Zhang C., Li J.H., Liu B., Chen H.W., Hu B., Luo Q., Zhang Y. (2026). Quantitative exploration of aging–precipitate–property relationship in Mg-Gd alloys. J. Mater. Sci. Technol..

[35] Liu S.J., Yang G.Y., Luo S.F., Jie W.Q. (2015). Microstructure evolution during heat treatment and mechanical properties of Mg–2.49Nd–1.82Gd–0.19Zn–0.4Zr cast alloy. Mater. Charact..

[36] Yan J.L., Sun Y.S., Xue F., Xue S., Xiao Y.Y., Tao W.J. (2009). Creep behavior of Mg–2wt.%Nd binary alloy. Mater. Sci. Eng. A.

[37] Yin D.D., Wang Q.D., Gao Y., Chen C.J., Zheng J. (2011). Effects of heat treatments on microstructure and mechanical properties of Mg–11Y–5Gd–2Zn–0.5Zr (wt.%) alloy. J. Alloys Compd..

